# *Lactobacillus plantarum* IOB602 and Its Postbiotics Attenuate Hypertension-Induced Damage by Modulating the RAS, PI3K/AKT/eNOS Pathways, and Gut Microbiota

**DOI:** 10.3390/foods15111869

**Published:** 2026-05-25

**Authors:** Yining Wang, Weilong Liu, Jingyang Tong, Chao Huo, Xuemei Han, Xuegang Luo

**Affiliations:** 1 Key Laboratory of Industrial Fermentation Microbiology of the Ministry of Education and Tianjin Key Laboratory of Industrial Microbiology, College of Biotechnology, Tianjin University of Science and Technology, Tianjin 300457, China; wyning620@163.com (Y.W.);; 2 Tianjin Key Laboratory of Edible Probiotics, Tianjin 300301, China

**Keywords:** hypertension, *Lactobacillus plantarum* IOB602, postbiotic, renin-angiotensin system, gut microbiota

## Abstract

Hypertension is a common cardiovascular disorder, and current pharmacological treatments are often associated with significant side effects, highlighting the need for safer alternatives. Probiotics and their postbiotics have emerged as promising candidates due to their favorable safety profiles. This study evaluated the potential of *Lactobacillus plantarum* IOB602 and its 602P postbiotic to attenuate hypertension-induced damage. We first assessed their ACE inhibitory activity in vitro. Subsequently, we investigated their protective effects against organ damage and the underlying mechanisms in L-NAME-induced hypertensive rats using biochemical assays, real-time qPCR, histopathological analysis, and 16S rRNA sequencing. In vitro results showed that IOB602 exhibited strong tolerance to simulated gastric acid and bile salts, indicating good gastrointestinal survivability. Both the culture supernatant and the postbiotic displayed significant ACE inhibitory activity, with the postbiotic achieving an inhibition rate of 82.21%. In vivo, treatment with IOB602 or 602P significantly reduced plasma angiotensin II levels, upregulated the PI3K-Akt-eNOS pathway, restored nitric oxide bioavailability, and attenuated oxidative stress and inflammatory responses in hypertensive rats. Histological analysis revealed that both interventions alleviated pathological damage in the thoracic aorta, heart, and kidneys. Furthermore, IOB602 and its postbiotic reshaped the gut microbiota composition by decreasing harmful genera such as *Ruminococcus* and enriching beneficial taxa including *Akkermansia* and *Christensenellaceae*. In conclusion, *L. plantarum* IOB602 and its 602P postbiotic show potential for development as functional foods or pharmaceutical adjuvants for the treatment of hypertension-induced organ damage.:

## 1. Introduction

Hypertension is a systemic, lifelong chronic disease characterised primarily by elevated arterial blood pressure in the systemic circulation [1]. As a major risk factor for numerous cardiovascular diseases, it is also one of the leading causes of global all-cause mortality and disability [2].This is primarily due to an imbalance between cardiac output and peripheral vascular resistance, typically driven by dysfunction of the renin-angiotensin-aldosterone system, excessive sympathetic nervous system activity, or impaired renal sodium excretion [3]. Persistent elevation of blood pressure predisposes individuals to target organ damage, with hypertensive heart disease being the most common manifestation [4]. Globally, the number of adults with hypertension has doubled, and the prevalence of hypertension is higher among men than among women [3]. Hypertension is also closely associated with a range of complications, including ischaemic heart disease, chronic kidney disease, cognitive impairment and metabolic syndrome [5,6,7,8]. Consequently, hypertension and its associated conditions pose a serious threat to individual health while also increasing the societal burden of cardiovascular disease and all-cause mortality. Modern medical research has confirmed that the pathogenesis of hypertension is significantly influenced by dietary patterns, particularly dietary habits such as high sodium intake, high saturated fat intake and insufficient dietary potassium intake [9]. Such dietary patterns promote vascular remodelling, endothelial dysfunction and persistent peripheral vasoconstriction, ultimately leading to long-term elevated blood pressure and gradually affecting organs such as the heart and kidneys [10]. In most cases, lifestyle interventions alone are insufficient to effectively mitigate the damage caused by hypertension. Furthermore, pharmacological treatment for hypertension is limited by the numerous contraindications, adverse reactions and high costs associated with traditional synthetic antihypertensive drugs [11]. These limitations highlight the importance of developing treatment strategies that are more cost-effective, safer and associated with lower toxicity and fewer side effects.

Recent studies have shown a significant association between hypertension and changes in the gut microbiota. Hypertension affects the integrity of the intestinal epithelium and the homeostasis of the gut microbiota [12]. The gut microbiota and its metabolites play a significant role in the regulation of blood pressure in humans; the gut microbiota is involved in the synthesis of short-chain fatty acids and the production of trimethylamine oxide, and influences the function of the intestinal mucosal barrier, thereby participating directly or indirectly in the regulation of blood pressure [13]. Persistent hypertension can disrupt the intestinal epithelial barrier and increase intestinal permeability, thereby inducing chronic low-grade inflammation in the gut and altering the composition of the gut microbiota [14]. These alterations in the gut microbiota lead to changes in microbiome-associated gene pathways in the host, which in turn elicit systemic inflammation and disrupt intestinal mechanotransduction. This, in turn, activates the renin–angiotensin–aldosterone system, the autonomic nervous system, and the immune system, ultimately exacerbating the damaging effects of hypertension [15].

With the continuous progress in gut microbiota research, studies utilising probiotics for the prevention or treatment of certain diseases are increasing. Probiotics are live microorganisms that, when administered in adequate amounts, confer a health benefit on the host [16]. Probiotics can colonize the human gut and reproductive tract. Through gut colonization, specific probiotic strains have been shown to prevent fatty liver disease, alleviate diabetes, and exert antioxidant effects [17,18]. Additionally, they may offer potential preventive benefits against hypertension [19]. Both epidemiological studies and animal experiments suggest that gut microbiota dysbiosis is closely associated with the development of hypertension [20]. In recent years, probiotic-fermented milk has been confirmed to inhibit the biological activity of angiotensin-converting enzyme (ACE) and exert antihypertensive effects in spontaneously hypertensive rats [21,22]. Postbiotics, defined as preparations of inanimate microorganisms and/or their components that confer a health benefit on the host [23], are more structurally stable and more easily absorbed by the human body compared to live strains [24]. Previous studies have shown that postbiotics exert a protective effect in various metabolic diseases by modulating the gut microbiota composition, improving intestinal barrier function, suppressing inflammatory responses, and regulating the renin-angiotensin system [25,26]. Therefore, postbiotics may represent a new strategy for alleviating damage to target organs associated with hypertension.

This study focused on *Lactiplantibacillus plantarum* IOB602 and evaluated the inhibitory effects of its culture supernatant and postbiotic on ACE activity in vitro. Using an L-NAME-induced hypertensive rat model, we measured organ indices, oxidative stress markers, inflammatory factors, and endothelial function. In addition, gut microbiome sequencing was performed to investigate the protective effects of live 602 and its 602P postbiotic on hypertensive rats, with the aim of establishing a theoretical foundation for the development of probiotics and functional postbiotic products that attenuate hypertension-induced damage.

## 2. Materials and Methods

### 2.1. Materials and Reagents

#### 2.1.1. Chemicals and Kits

N-[3-(2-Furyl)acryloyl]-L-phenylalanyl-glycyl-glycine (FAPGG) and angiotensin-converting enzyme (ACE) were purchased from MedChemExpress (MCE, Monmouth Junction, NJ, USA). Hydrochloric acid, bovine bile salt, and sodium chloride were purchased from Tianjin Jiangtian Chemical Technology Co., Ltd. (Tianjin, China). Nω-Nitro-L-arginine methyl ester (L-NAME) was purchased from Shanghai Macklin Biochemical Co., Ltd. (Shanghai, China). Trizol reagent was purchased from Thermo Fisher Scientific (Waltham, MA, USA). Reverse transcription kit and fluorescence quantitative PCR kit were purchased from Beijing TransGen Biotech Co., Ltd. (Beijing, China). Superoxide dismutase (SOD), glutathione (GSH), malondialdehyde (MDA), and nitric oxide (NO) assay kits were purchased from Nanjing Jiancheng Bioengineering Institute (Nanjing, China). Endothelial nitric oxide synthase (eNOS), angiotensin II (Ang II), interleukin-6 (IL-6), and interleukin-1β (IL-1β) ELISA kits were purchased from Shanghai Zhuocai Biotechnology Co., Ltd. (Shanghai, China).

#### 2.1.2. Probiotic Strain

*L. plantarum* IOB602 was isolated from naturally fermented sourdough and is deposited at the China General Collection of Microorganisms (CGMCC No. 16021). It has a viable count of 6.5 × 10^11^ CFU/g and was supplied by Tianjin Chuangyuan Biotechnology Co., Ltd. (Tianjin Key Laboratory of Edible Probiotics, Tianjin, China).

### 2.2. Instruments and Equipment

The clean bench (SW-CJ-2FD) was supplied by Suzhou Antai Air Technology Co., Ltd. (Sujing Group, Suzhou, China). The full-wavelength microplate reader (SpectraMax 190) was supplied by Molecular Devices (Sunnyvale, CA, USA). The microcentrifuge (Sorvall™ Legend™ Micro 17R) was supplied by Thermo Fisher Scientific (Waltham, MA, USA). The tissue homogenizer (TP-24) was supplied by Jieling Instrument Manufacturing (Tianjin) Co., Ltd. (Tianjin, China). The real-time fluorescence quantitative PCR instrument (QuantStudio 1 Plus) was supplied by Thermo Fisher Scientific (Waltham, MA, USA).

### 2.3. Determination of the Growth Curve

*L. plantarum* IOB602 was activated twice and inoculated into MRS liquid medium, incubated at 37 °C over 24 h. Samples were taken every 1 h to measure the OD_600_, and the growth curve was plotted.

### 2.4. Determination of Acid and Bile Salt Tolerance

Acid tolerance assay: The *L. plantarum* IOB602 strain after 12 h of incubation was inoculated into MRS liquid medium with pH values of 1.0, 2.0, 3.0, and 4.0. Bile salt tolerance assay: *L. plantarum* IOB602 was inoculated into MRS liquid medium containing bile salt concentrations of 0.1%, 0.3%, and 0.5%. MRS liquid medium without bile salt and at a conventional pH was used as the negative control group. After incubation at 37 °C for 4 h, the OD_600_ was measured to compare growth status. The survival rate was calculated using Equation (1)

(1) $$Survival\;rate\;=\;\frac{A}{B}\;\times \;100\%$$

A—OD_600_ absorbance value of the experimental group, Abs;

B—OD_600_ absorbance value of the control group, Abs.

### 2.5. Preparation of L. plantarum IOB602 Postbiotics

*L. plantarum* IOB602 was activated and inoculated into MRS liquid medium for fermentation. After the bacteria entered the stationary phase, the culture was centrifuged to collect the pellet, which was inactivated at 95 °C, followed by freeze-drying. Inactivation was confirmed by plating on MRS agar and incubating aerobically at 37 °C for 48 h; no viable colonies were detected. The postbiotic powder was standardized to a viable count equivalent of 6.5 × 10^11^ CFU/g based on the original viable count of the IOB602 strain before inactivation. The resulting postbiotics were packaged and stored at −20 °C.

### 2.6. Determination of ACE Inhibition Rate

The ACE inhibition rate was determined with reference to the method of Shalaby et al. [27] with modifications. The specific steps are as follows:

Preparation of working solutions: FAPGG was dissolved in Tris-HCl buffer, and ACE was dissolved in sterile nuclease-free water to prepare 0.88 mmol/L FAPGG and 0.25 U/mL ACE working solutions, respectively.

Sample preparation: Twice-activated *L. plantarum* IOB602 was inoculated into MRS liquid medium and cultured at 37 °C for 24 h, centrifuged at 7000× *g* for 10 min, and the supernatant was filtered through a 0.22 µm microporous membrane for later use. Using a gradient counting method, postbiotics equivalent to the same concentration as the bacterial suspension were dissolved in liquid MRS medium for later use.

150 µL of FAPGG working solution was added to a transparent 96-well plate, pre-incubated at 37 °C for five minutes. Then, we added 10 µL of ACE working solution and 10 µL of the sample, mixed thoroughly by shaking, and immediately measured the OD340. After incubation at 37 °C for 15 min, the OD_340_ was measured again. The same procedure was applied to the no-enzyme control group B (ACE working solution replaced with sterile nuclease-free water), the enzyme control group C (sample replaced with Tris-HCl), and the blank control group D (both ACE working solution and sample replaced with sterile nuclease-free water and Tris-HCl, respectively). The specific reaction system is shown in Table 1. The ACE inhibition rate was calculated using Equation (2).

The ACE inhibition rate was calculated in vitro using Equation (2).

(2) $$\mathrm{ACE}\;\mathrm{inhibition}\;\mathrm{rate}=\frac{\left\{[(C_{1}\;-C_{2})\;-\;(D_{1}\;-\;D_{2})]\;-\;[(A_{1}\;-\;A_{2})\;-\;(B_{1}\;-\;B_{2})]\right\}}{\left[\left(C_{1}\;-\;C_{2})\;-\;(D_{1}\;-\;D_{2}\right)\right]}\;\times \;100\%$$

A_1_—OD_340_ nm absorbance value of sample group A before the 15-min incubation, Abs;

A_2_—OD_340_ nm absorbance value of sample group A after the 15-min incubation, Abs;

B_1_—OD_340_ nm absorbance value of the no-enzyme control group B before the 15-min incubation, Abs;

B_2_—OD_340_ nm absorbance value of the no-enzyme control group B after the 15-min incubation, Abs;

C_1_—OD_340_ nm absorbance value of ACE control group C before the 15-min incubation, Abs;

C_2_—OD_340_ nm absorbance value of ACE control group C after the 15-min incubation, Abs;

D_1_—OD_340_ nm absorbance value of blank control group D before the 15-min incubation, Abs;

D_2_—OD_340_ nm absorbance value of blank control group D after the 15-min incubation, Abs.

### 2.7. Animal Grouping and Administration

Five-week-old specific pathogen-free (SPF) male Wistar rats (weighing 150–180 g, license No. SCXK (Jing) 2019-0010) were obtained from Sibefu Biotechnology Co., Ltd. (Beijing, China). All animals were acclimated to standard conditions (24 ± 2 °C, 50 ± 5% humidity, 12/12 h light/dark cycle) for one week prior to the experiment, during which they were free to consume food and water. The study was approved by the Animal Care and Ethics Committee of Tianjin University of Science and Technology (approval No. SWXY-20240314108).

After one week of acclimation, a total of 50 rats were used. Ten rats were randomly selected as the normal control group (NC). The remaining 40 rats received drinking water containing L-NAME (400 mg/L) for 8 weeks to induce hypertension. At week 4 of L-NAME administration, blood samples were collected from the retro-orbital sinus to measure plasma NO levels. Rats showing a significant reduction in NO levels compared with NC group were considered successfully hypertensive and then randomly assigned into four groups (*n* = 10 per group): model group (M), positive control group (PC), IOB602 probiotic group (602), and IOB602 postbiotic group (602P). From week 5 to week 8 (the remaining 4 weeks), the PC group received captopril (10 mg/kg/day) by gavage. The 602 and 602P groups received 1 × 10^9^ CFU/kg/day of live IOB602 or its postbiotic by gavage, respectively. The M group received an equivalent volume of physiological saline by gavage. The NC group continued to receive normal drinking water throughout the 8-week period.

### 2.8. Animal Sample Collection and Processing

After four weeks of intervention, rats in each group were weighed, blood was collected, and the thoracic aorta, kidneys, and heart were harvested. Blood was placed in heparin anticoagulant tubes, centrifuged at 4 °C, 3500 r/min for 10 min to obtain plasma samples. Various physiological and biochemical indicators were detected according to the kit instructions. The heart and kidneys were quickly weighed. Subsequently, parts of the left ventricle of the heart, kidneys, and the distal thoracic aorta were immersed in 4% paraformaldehyde solution for fixation to prepare tissue sections. Prepared sections were stained with HE and observed under a microscope at 200× (thoracic aorta) and 400× (heart, kidney) magnification for photography. The remaining heart tissue was rinsed with cold sterile physiological saline, flash-frozen in liquid nitrogen, and stored at −80 °C. Cardiac injury was determined based on the following parameters: cardiomyocyte hypertrophy, myocardial fiber disarray, inflammatory cell infiltration, and interstitial edema. Each parameter was graded on a 0–4 scale (0 = absent, 1 = mild, 2 = moderate, 3 = severe, 4 = very severe), and the scores were summed to obtain a total cardiac injury score (maximum 16). Thoracic aortic injury was assessed using the following criteria: endothelial integrity, intimal-medial thickening, and smooth muscle cell disorganization, each graded on the same 0–4 scale and summed to yield a total aortic injury score (maximum 12). For each group, five random fields per heart and per aortic section were examined, and the average scores were used for statistical analysis.

### 2.9. RT-qPCR Analysis

Rat myocardial tissue stored at −80 °C was placed in sterile nuclease-free centrifuge tubes. cDNA obtained from total RNA that had been extracted with the Trizol method was subjected to fluorescence quantitative PCR under the following conditions: pre-denaturation at 95 °C for 2 min, denaturation at 95 °C for 10 s, annealing at 60 °C for 30 s (cycle number: 40), extension at 95 °C for 1 min, 55 °C for 1 min, and 95 °C for 15 s. Relative expression of target genes was assessed using the 2^−ΔΔCT^ method. The primer sequences used are listed in Table 2.

### 2.10. Gut Microbiota Analysis

Following collection from rats, colon contents were cryopreserved at −80 °C and subsequently transported to Shanghai Sanshu Biotechnology Co., Ltd. (Shanghai, China) for 16S rDNA sequencing. Genomic DNA extraction was followed by amplification of the V3–V4 region of bacterial 16S rDNA with primer pair 338F (5′-ACTCCTACGGGGAGGCAGCAG-3′) and 806R (5′-GGACTACHVGGGTWTCTAAT-3′). The Illumina NovaSeq 6000 SP Reagent Kit enabled high-throughput sequencing, and the DADA2 model was applied for amplicon sequence variant (ASV) classification. After quality control and chimera removal, an average of 211,516 clean reads per sample was obtained, with an average raw read count of 224,859.

### 2.11. Data Processing

Each experiment was repeated in triplicate, and experimental data are expressed as “Mean ± Standard Deviation (SD)”. Measurement data were analysed by one-way analysis of variance (ANOVA) and inter-group tests using GraphPad Prism 10.4.1software. In the animal study, after 4 weeks of modelling, rats were randomly assigned to four groups (*n* = 10 per group). During administration and sample collection, the experimenters were blinded to group allocation to minimize bias.

## 3. Results and Discussion

### 3.1. Identification and Biochemical Properties of L. plantarum IOB602

**Figure 1:**
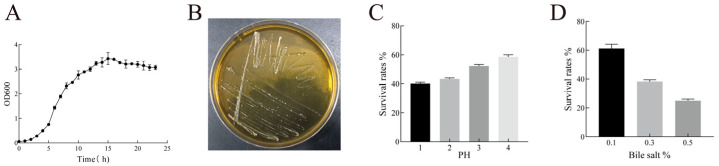
Strain identification and biochemical properties analysis of *L. plantarum* IOB602. (**A**) Growth Curve of *L. plantarum* IOB602; (**B**) Colony morphology of L. plantarum IOB602; (**C**) Survival rate of *L. plantarum* IOB602 at different pH levels; (**D**) Survival rate of *L. plantarum* IOB602 with different concentrations of bile salts.

The growth kinetics of *Lactiplantibacillus plantarum* IOB602 are shown in Figure 1A. The strain initiated logarithmic growth after 3 h and entered the stationary phase at 16 h, attaining maximum cell density. On MRS solid agar, IOB602 formed small, white, spherical colonies (Figure 1B). A prerequisite for probiotics to exert beneficial effects in vivo is their ability to survive the harsh gastrointestinal environment, where gastric pH is approximately 3.0 and small intestinal bile salt concentration is around 0.3% [28]. To assess this potential in *L. plantarum* IOB602, we evaluated its tolerance to simulated gastric acid and bile salts.

After exposure to pH 1, 2, 3, and 4, the viability of 602 was 40.07%, 43.37%, 52.20%, and 58.63%, respectively (Figure 1C). Following a 4 h incubation with 0.1%, 0.3%, and 0.5% bile salts, the survival rates were 61.20%, 38.29%, and 25.13%, respectively (Figure 1D). Together, these findings demonstrate that *L. plantarum* IOB602 exhibits substantial acid and bile salt tolerance, indicating a robust capacity to survive gastrointestinal transit and potentially colonise the intestine.

### 3.2. ACE Inhibitory Activity of L. plantarum IOB602 Culture Supernatant and Postbiotic

**Figure 2:**
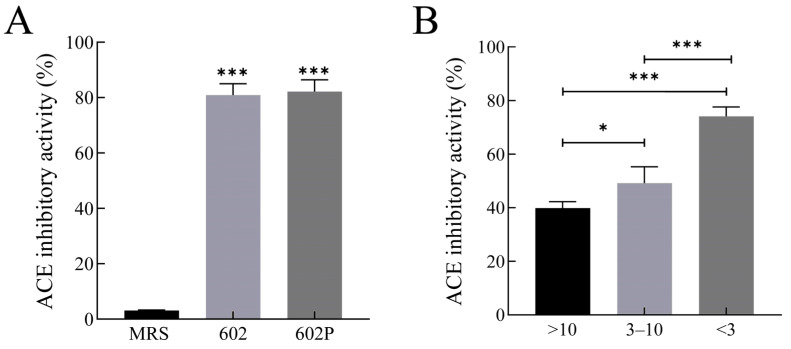
ACE inhibitory activity of *L. plantarum* IOB602. (**A**) Comparison between culture supernatant and postbiotic (**B**) Ultrafiltered fractions derived from the culture supernatant, separated by molecular weight cutoffs (>10 kDa, 3–10 kDa, and <3 kDa). Data are expressed as mean ± SD. Statistical significance is indicated as * *p* < 0.05 and *** *p* < 0.001. MRS refers to the sterile MRS liquid medium used as a negative control. 602 denotes the *L. plantarum* IOB602 culture supernatant, while 602P represents its postbiotic. The fractions obtained after ultrafiltration are labeled as >10 (retentate > 10 kDa), 3–10 (fraction between 3 and 10 kDa), and <3 (filtrate < 3 kDa).

Angiotensin-converting enzyme (ACE) is widely distributed in human tissues and body fluids, where it plays a central role in blood pressure regulation. This enzyme catalyzes the conversion of angiotensin I into the potent vasopressor angiotensin II, while simultaneously degrading bradykinin, a nonapeptide with vasodilatory properties [29]. Through this dual mechanism, ACE activity promotes vasoconstriction and elevates systemic blood pressure. To determine whether *L. plantarum* IOB602 might alleviate hypertension-induced damage by influencing ACE, we assessed the ACE inhibitory activity of its culture supernatant and postbiotic. As shown in Figure 2A, both preparations exhibited significantly higher ACE inhibition rates than the negative control (MRS liquid medium, *p* < 0.001). The postbiotic achieved an inhibition rate of 82.21 ± 4.28%, compared to 80.92 ± 4.10% for the culture supernatant. The difference did not reach statistical significance, indicating that the primary ACE-inhibitory activity resides in the extracellular metabolites secreted by IOB602, rather than being retained within the bacterial cells. To further characterize the nature of the ACE-inhibitory metabolites produced by IOB602, we subjected the culture supernatant to ultrafiltration. This process yielded three molecular weight fractions: 10–30 kDa, 3–10 kDa, and below 3 kDa. When we tested each fraction for ACE inhibitory activity, a clear pattern emerged (Figure 2B). The <3 kDa fraction demonstrated significantly higher inhibition than either of the larger molecular weight fractions (*p* < 0.001), indicating that the primary active components are low molecular weight metabolites. Interestingly, all three fractions retained considerable ACE inhibitory capacity. Even the 10–30 kDa fraction, despite containing the largest molecules, showed activity as high as 39.89%. We infer that IOB602 produces multiple ACE-inhibitory metabolites, which differ substantially in their molecular weights. Since each fraction retained substantial activity on its own and the unfractionated supernatant achieved 80.92%, we elected to continue subsequent experiments using the whole bacterial culture.

### 3.3. Effects of L. plantarum IOB602 and Its Postbiotic on Organ Coefficients and Endothelial Function in Hypertensive Rats

**Figure 3:**
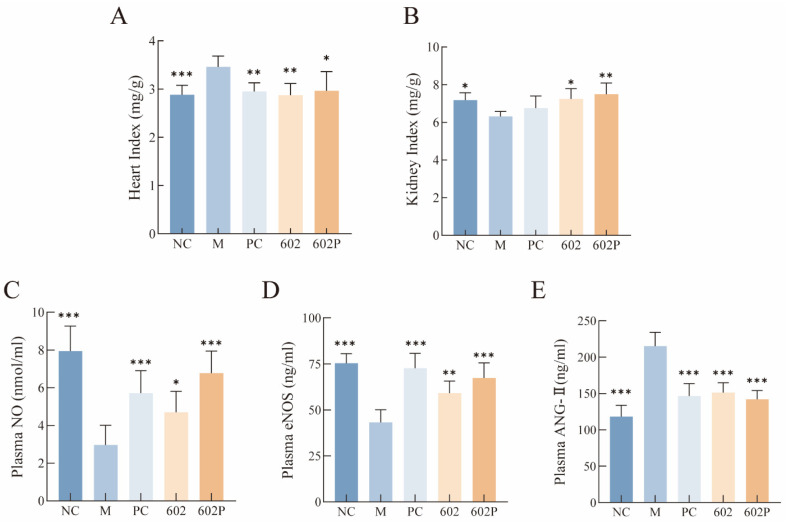
Influence of *L. plantarum* IOB602 and its 602P postbiotic on signs and physiological and biochemical indices of hypertensive rat. (**A**) Heart index; (**B**) Kidney index; (**C**) Plasma NO level; (**D**) Plasma eNOS level; (**E**) Plasma ANG-II level. Data are presented as the mean ± SD. * *p* < 0.05, ** *p* < 0.01, *** *p* < 0.001 than the M group.

Following the 4-week intervention, all rats were euthanized for tissue collection. Final body weights were recorded, and the hearts and kidneys were excised and weighed to calculate the respective organ indices (organ-to-body weight ratios). As shown in Figure 3A, the cardiac index was significantly elevated in the hypertensive model group compared to the normotensive controls (*p* < 0.001), indicating compensatory cardiac hypertrophy likely due to sustained pressure overload. Treatment with *L. plantarum* IOB602, 602P postbiotic, or the drug captopril significantly attenuated this increase in heart-to-body weight ratio, confirming a protective effect against hypertension-induced cardiac remodelling. In contrast, the renal index was markedly reduced in the model group relative to controls (*p* < 0.05, Figure 3B). Both the probiotic and postbiotic treatments attenuated this reduction, suggesting partial protection against L-NAME-induced renal pathology, including fibrosis, atrophy, and apoptosis.

The renin-angiotensin system (RAS), primarily mediated through the ACE-Ang II-AT1R axis, plays a critical role in blood pressure regulation [30]. Its key effector peptide, Angiotensin II (Ang II), drives hypertensive pathophysiology by promoting vasoconstriction, sodium retention, inflammation, oxidative stress, and proliferation of vascular smooth muscle cells and cardiomyocytes [31,32,33]. Conversely, vascular endothelial function is reliably indicated by the activity of eNOS and the bioavailability of NO. Located within endothelial cells, eNOS catalyzes the conversion of L-arginine to L-citrulline to generate NO, a potent signaling molecule that induces vascular relaxation and inhibits smooth muscle cell proliferation [34,35].

To explore how *L. plantarum* IOB602 and its postbiotic protect vascular endothelium in L-NAME-induced hypertension, we measured plasma levels of NO, eNOS, and Ang II (Figure 3C–E). Compared to normol controls, model group rats showed significantly lower NO and eNOS levels (*p* < 0.001), indicating suppression of the vasodilatory pathway. Intervention with either the viable IOB602 probiotic or its postbiotic significantly elevated these levels relative to the model group. Concurrently, plasma Ang II activity was markedly elevated in the model group, reaching 215.14 ng/mL (*p* < 0.001), reflecting marked RAS activation. The IOB602 probiotic, its postbiotic, and captopril all effectively reduced Ang II activity compared to the model group. Moreover, the postbiotic achieved the most pronounced reduction, lowering Ang II to 142.16 ng/mL (*p* < 0.001). In summary, these results point to a dual mechanism by which *L. plantarum* IOB602 and its postbiotic confer vascular protection. They appear to inhibit ACE activity, thereby dampening aberrant RAS activation and Ang II overproduction, while simultaneously counteracting L-NAME-induced eNOS suppression to restore NO bioavailability, but further validation is needed.

### 3.4. L. plantarum IOB602 and Its Postbiotic Reduce Oxidative Stress and Inflammatory Response in Hypertensive Rats

**Figure 4:**
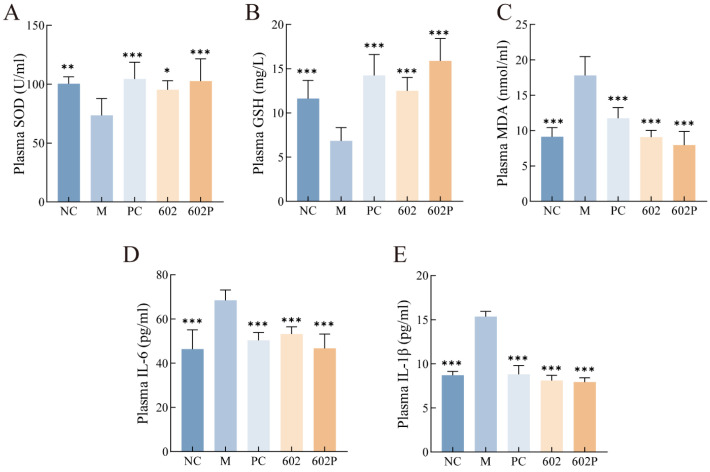
Effects of *L. plantarum* IOB602 and its 602P postbiotic on oxidative stress and inflammation in hypertensive rats. (**A**) Plasma SOD level; (**B**) Plasma GSH level; (**C**) Plasma MDA level; (**D**) Plasma IL-6 level; (**E**) Plasma IL-1β level. Data are presented as the mean ± SD. * *p* < 0.05, ** *p* < 0.01, *** *p* < 0.001 than the M group.

Oxidative stress, resulting from an imbalance between reactive oxygen species (ROS) production and antioxidant defencess, serves as a central factor in the pathogenesis of endothelial dysfunction in hypertension. Specifically, ROS contribute to vascular pathology by inactivating nitric oxide and directly inducing vasoconstriction and vascular fibrosi [36]. In our model, L-NAME administration significantly reduced plasma levels of superoxide dismutase (SOD) and glutathione (GSH), while markedly increasing malondialdehyde (MDA) content (*p* < 0.001) (Figure 4A–C). These alterations confirm that chronic NO depletion induces a state of oxidative stress. Intervention with the IOB602 probiotic substantially restored antioxidant capacity, elevating SOD from 73.45 U/mL to 95.23 U/mL and GSH from 6.83 mg/L to 12.48 mg/L (*p* < 0.001). The postbiotic produced an even more pronounced increase, raising these values to 102.70 U/mL and 15.87 mg/L (*p* < 0.001). Furthermore, both treatments also effectively curbed lipid peroxidation, reducing plasma MDA content from 17.79 nmol/mL to 9.08 nmol/mL and 7.95 nmol/mL.

**Figure 5:**
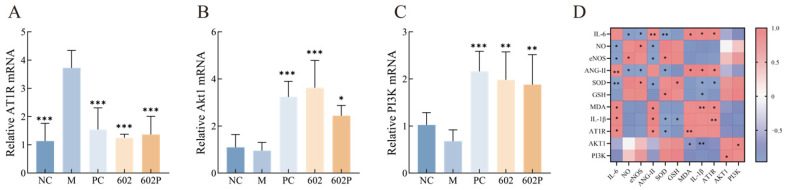
Effects of *L. plantarum* IOB602 and its postbiotic on mRNA levels of AT1R (**A**), Akt1 (**B**), and PI3K (**C**) in heart. (**D**) Correlation analysis of the correlation between cardiac mRNA transcription and plasma biochemistry. Data are presented as the mean ± SD. * *p* < 0.05, ** *p* < 0.001, *** *p* < 0.001 than the M group.

Previous studies have shown that Ang II is a key driver of ROS production via NADPH oxidase activation, and elevated ROS levels can lead to excessive scavenging of NO, thereby impairing vasodilation and ultimately increasing peripheral vascular resistance that amplifies both hypertension-related and oxidative damage [37,38]. In this study, we observed that IOB602 and its postbiotic significantly reduced plasma Ang II levels, downregulated cardiac AT1R expression, and increased plasma NO levels (Figure 3C,E and Figure 5A). Concurrently, IOB602 and its postbiotic effectively enhanced the host’s antioxidant system, scavenged peroxides, and mitigated lipid peroxidation damage. We hypothesize that IOB602 reduces ROS production by inhibiting NADPH oxidase activation downstream of Ang II/AT1R signaling. At the same time, its enhanced antioxidant capacity helps scavenge residual ROS and reduce NO scavenging, although further validation is required.

The relationship among oxidative stress, inflammatory responses, and hypertension has been widely concerned [38]. For example, ROS can activate the NLRP3 inflammasome, promoting the release of pro-inflammatory cytokines such as IL-1β and IL-18, which leads to vascular damage [39]. Beyond this direct inflammasome pathway, IL-6 engages its downstream signaling cascades to form a cross-talk with ROS-driven oxidative stress, thereby amplifying the pathological processes that accelerate hypertension progression [40]. To evaluate the immunomodulatory effects of 602 and its 602P postbiotic in hypertensive rats, we measured plasma levels of the pro-inflammatory cytokines IL-6 and IL-1β (Figure 4D,E). Compared with rats in the NC group, plasma IL-6 and IL-1β levels were significantly elevated in the M group (*p* < 0.001). This elevation suggests that L-NAME-induced hypertension promotes an inflammatory response, likely via oxidative stress pathways that drive the release of pro-inflammatory cytokines including IL-6 and IL-1β. Treatment with 602 probiotic, 602P postbiotic, or captopril all significantly reduced plasma IL-6 and IL-1β levels relative to the model group (*p* < 0.001). Notably, the probiotic and postbiotic achieved effects comparable to those of captopril, underscoring their potential as anti-inflammatory interventions. These findings indicate that both live 602 and its postbiotic can modulate levels of inflammatory factors, thereby alleviating the tissue damage caused by hypertension in rats.

### 3.5. L. plantarum IOB602 and Its Postbiotic Regulate Gene Expression Related to Blood Pressure Control

The PI3K-Akt-eNOS signaling pathway plays a crucial role in regulating vascular endothelial function and blood pressure. Endothelial dysfunction, a key factor in hypertension pathogenesis, is closely linked to dysregulation of this pathway [41]. Activation of PI3K-Akt-eNOS alleviates oxidative stress by enhancing antioxidant enzyme expression and reducing reactive oxygen species generation. It also suppresses pro-inflammatory factors such as IL-6, IL-1β, and TNF-α, thereby mitigating inflammatory responses and helping to preserve vascular function and limit end-organ damage [42]. Within the renin-angiotensin system, Angiotensin II exerts its effects primarily by binding to the AT1 and AT2 receptors. The AT1 receptor mediates most of the pathophysiological actions of Ang II, including vasoconstriction, sodium and water reabsorption, and the promotion of oxidative stress and inflammatory responses [43]. Through this receptor, Ang II binding induces vascular smooth muscle contraction and increases peripheral vascular resistance, representing a key mechanism driving hypertension progression [44].

To elucidate the mechanisms underlying the attenuation of hypertension-induced damage and cardioprotective effects of 602 and its 602P postbiotic in L-NAME-induced hypertensive rats, we harvested cardiac tissue after four weeks of treatment and examined the expression of PI3K, AKT1, and AT1R by real-time qPCR (Figure 5). In the model group, PI3K and AKT1 expression in cardiac tissue decreased relative to controls. Conversely, AT1R expression showed a marked upregulation, increasing approximately three-fold (*p* < 0.001). Following intervention with 602 or its 602P postbiotic, this pattern was reversed: PI3K and AKT1 were significantly upregulated, and AT1R was downregulated, compared to the model group (*p* < 0.001). These findings point to a dual mechanism by which 602 and its 602P postbiotic may confer cardiovascular protection. On one hand, they appear to suppress excessive RAS activation by downregulating AT1R expression, thereby inhibiting vascular smooth muscle cell proliferation, cardiomyocyte hypertrophy, and myocardial fibrosis. This is consistent with previous reports showing that *L. plantarum* supplementation can attenuate hypertension-induced cardiac hypertrophy and kidney damage by downregulating AT1R expression and modulating RAS signaling [45]. On the other, upregulation of the PI3K-Akt pathway may promote eNOS expression and NO production (Figure 3C,D), suppress oxidative stress and inflammation, and ultimately alleviate hypertension-induced vascular and cardiac damage. Previous studies have indicated that milk fermented by *L. plantarum* SR37-3 and SR61-2 has been shown to significantly reduce ANG-II levels in serum, alongside marked reductions in IL-6, TNF-α, and IL-1β, suggesting that *L. plantarum* fermentation products contribute to their anti-inflammatory and hypotensive effects [46]. Moreover, *L. fermentum* CECT5716 and *L. bacillus* CECT5711 have been shown to attenuate renal damage and suppress hypertension in spontaneously hypertensive rats [47], with additional evidence pointing to modulation of aortic Th17 cells by *L. fermentum* CECT5716 [48]. Pearson correlation analysis further indicated that plasma Ang II levels were significantly positively correlated with myocardial AT1R expression, whilst plasma IL-6, IL-1β and MDA were significantly positively correlated with Ang II and AT1R (*p* < 0.05); conversely, SOD was significantly negatively correlated with Ang II and AT1R (*p* < 0.05). These correlations provide integrated statistical support linking systemic RAS activation to the inflammatory and oxidative damage observed in hypertensive rats.

### 3.6. L. plantarum IOB602 and Its Postbiotic Attenuate Thoracic Aorta, Heart and Kidney Injury

**Figure 6:**
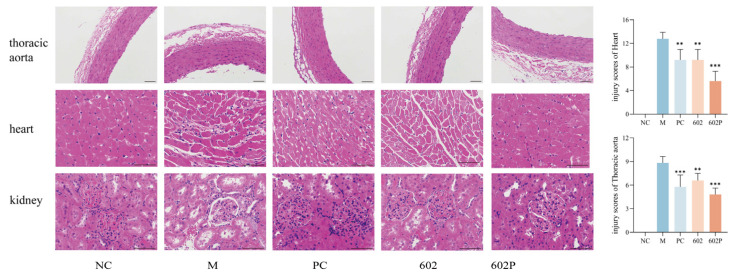
Representative micrographs of H&E-stained rats thoracic aorta tissue (×200), heart tissue and kidney tissue sections (×400). Scale bars, 200 μm. ** *p* < 0.001, *** *p* < 0.001 than the M group.

Histological analysis revealed that L-NAME induced histopathological damage in multiple organs of rats, which was attenuated by treatment with 602 or its 602P postbiotic (Figure 6). The control group had a smooth aortic endothelium with intact cells. The model group developed uneven wall thickness, intimal and medial hyperplasia, and endothelial damage—all attenuated by captopril, 602, or its 602P postbiotic, which restored normal vascular structure. In the control group, cardiomyocytes exhibited normal size and orderly arrangement. The model group, however, showed cardiomyocyte hypertrophy, tissue edema, disorganized architecture, inflammatory infiltration, and thickened myocardial fibers with signs of rupture. Intervention with 602 probiotic, 602P postbiotic, or captopril markedly improved myocardial fiber compactness and organization while reducing edema. Notably, the postbiotic exerted a significantly greater protective effect than either the probiotic or captopril. Renal histopathological examination showed uniform glomeruli and well-defined tubules in the control group. In contrast, the model group exhibited dilated glomerular capillaries, enlarged tubular lumens, and congested interstitial vessels. Treatment with captopril, 602, or its 602P postbiotic attenuated these L-NAME-induced renal structural abnormalities, conferring significant organ protection.

### 3.7. L. plantarum IOB602 and Its Postbiotic Ameliorate Gut Microbiota Dysbiosis in Hypertensive Rats

**Figure 7:**
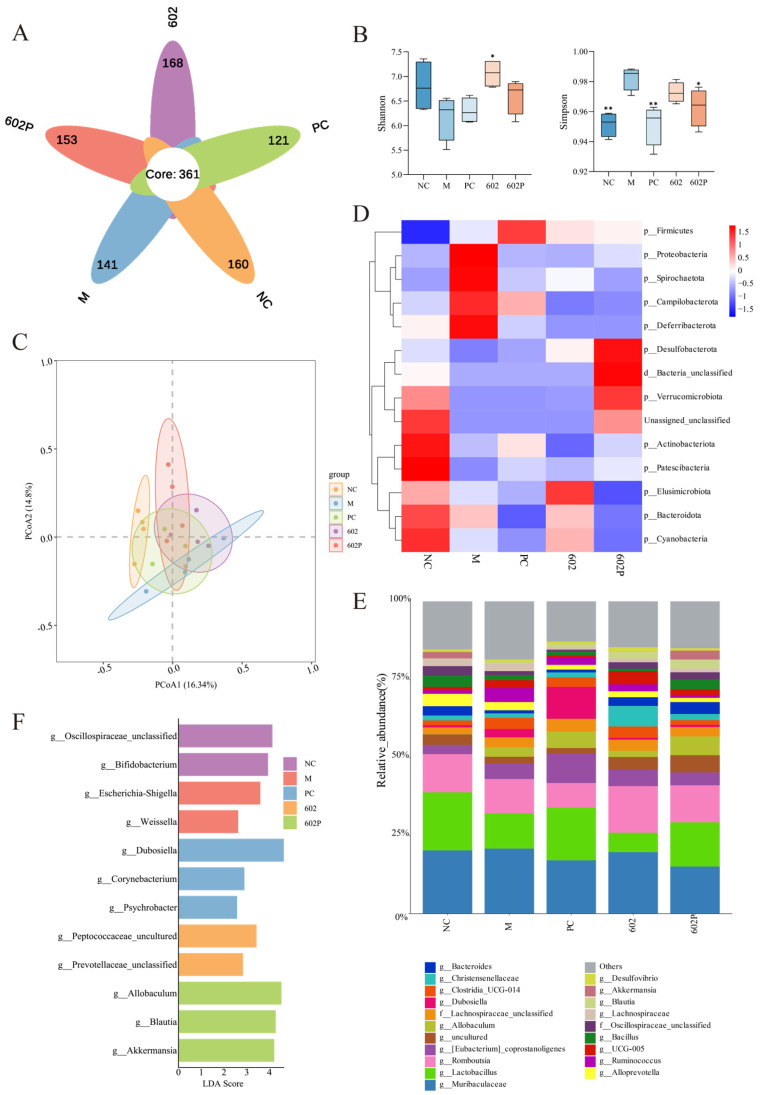
*L. plantarum* IOB602 and Its 602P postbiotic restores gut flora dysbiosis triggered by hypertensive. (**A**) Petal plot showing operational taxonomic units; (**B**) Alpha diversity presented as the index of Shannon and Simpson index; (**C**) Principal coordinate analysis analyzing β-diversity; (**D**) Species composition at the Phylum level; (**E**) Species composition at the Genus level; (**F**) LEfSe analysis shows taxa with substantial variances in abundance. * *p* < 0.05, ** *p* < 0.01 than the M group.

The gut microbiota is believed to play a significant role in the development of hypertension [49], with studies showing that *L. plantarum* CCFM639 reduces blood pressure in hypertensive mice by reducing levels of the harmful bacterium *S. aureofaciens* in the intestines [50]. To investigate whether *L. plantarum* IOB602 and its postbiotic modulate the gut microbiota in hypertensive rats, we performed 16S rRNA sequencing on colonic contents. Bioinformatic analysis revealed that both IOB602 and its postbiotic restored hypertension-induced alterations in gut microbial composition. As shown in the petal plot (Figure 7A), the distribution of microbial communities varied across groups. Shannon and Simpson analyses indicate that 602 and 602P can mitigate the reduction in community diversity caused by hypertension (Figure 7B). To assess differences in gut microbial composition, PCoA was performed. The analysis revealed distinct clustering between the NC and M groups. Treatment with 602 or 602P partially shifted the microbial clusters away from the M group, with the 602P group showing greater separation from M cluster and closer proximity to NC cluster (Figure 7C). These findings suggest that 602 and 602P can ameliorate hypertension-associated gut dysbiosis by modulating microbial community structure.

At the phylum level, hypertensive rats exhibited increased relative abundances of Proteobacteria, Spirochaetota, Campylobacterota, and Deferribacterota compared to the NC group, while Desulfobacterota, Verrucomicrobiota, and Patescibacteria were reduced (Figure 7D). Elevated Campylobacterota levels are closely associated with cardiovascular disease development and promote inflammatory responses through immune modulation [51,52]. Both 602 and its 602P postbiotic partially reversed these hypertension-induced shifts. Additionally, the 602 group showed increased Elusimicrobiota abundance, while the postbiotic group exhibited elevated Desulfobacterota levels. Analysis at the genus level revealed distinct compositional shifts across the five experimental groups (Figure 7E). *Ruminococcus*, a genus associated with intestinal inflammation and Crohn’s disease, is known to be enriched in hypertensive individuals [53,54]. Compared with the M group, the 602 and 602P groups showed a marked reduction in the relative abundance of *Ruminococcus*, with the 602P group returning to levels comparable to those of the NC group. Conversely, *Christensenellaceae*, which is enriched in healthy individuals and negatively correlated with cardiovascular disease risk [55], showed increased abundance in the 602 group. *Akkermansia*, a beneficial genus that promotes colonic mucus regeneration and enhances immune homeostasis, was significantly elevated in the 602P group relative to all other groups [56]. These findings demonstrate that *L. plantarum* IOB602 and its postbiotic, particularly the latter, effectively restored beneficial genera and suppressed harmful ones, underscoring their positive regulatory effect on gut microbial composition.

LEfSe analysis identified functionally distinct microbiota in each treatment group (Figure 7F). Based on LDA scores, *Escherichia-Shigella* and *Weissella* were significantly enriched in the M group, with the former known to be associated with hypertensive populations [57]. The 602 group showed specific enrichment of *Peptococcaceae* and *Prevotellaceae*, while *Allobaculum*, *Blautia*, and *Akkermansia* were significantly enriched in the postbiotic group. The enrichment of *Akkermansia* observed in the 602 and 602P groups may contribute to blood pressure reduction and organ protection through multiple interconnected pathways. Increased *Akkermansia* abundance has been shown to restore intestinal serotonin levels, thereby alleviating hypertension [58]. Moreover, *Akkermansia* can enhance regulatory T cell populations, which in turn reduce pro-inflammatory cytokine production and mitigate hypertensive damage [59]. In the present study, postbiotic treatment significantly reduced plasma IL-6 and IL-1β levels, which is consistent with an *Akkermansia*-mediated anti-inflammatory mechanism. *Allobaculum* and *Blautia*, significantly enriched in the postbiotic group according to LEfSe analysis, produce short-chain fatty acids (SCFAs) that exert anti-inflammatory and anti-oxidative effects [60,61]. *Blautia* also modulates aldosterone, a key hormone in the renin-angiotensin-aldosterone system [62]. SCFAs activate the PI3K-Akt-eNOS pathway via G-protein-coupled receptors and upregulate ACE2 to restrain excessive RAS activation [63]. This is consistent with our observations of restored NO bioavailability, elevated superoxide dismutase and glutathione, and reduced malondialdehyde content. Collectively, gut microbiota remodeling by IOB602 and its postbiotic, particularly the enrichment of *Akkermansia*, *Allobaculum*, and *Blautia*, likely acts upstream of the RAS-oxidative stress-inflammation axis, attenuating hypertension-induced damage through SCFA/GPCR signaling, Treg-mediated anti-inflammation, and enhanced NO bioavailability. It should be noted that these microbiota changes remain associative, and their causal role awaits validation in future experiments.

## 4. Conclusions

In this study, we demonstrated that *L*. *plantarum* IOB602 and its postbiotic (602P) exert protective effects against hypertension-induced damage in L-NAME-treated rats. Both interventions inhibited ACE activity in vitro, reduced plasma angiotensin II levels in vivo, and restored NO bioavailability via upregulation of the PI3K-Akt-eNOS pathway. They also attenuated oxidative stress and inflammation, while alleviating histopathological damage in the aorta, heart, and kidneys. Furthermore, 602 and 602P reshaped the gut microbiota composition by decreasing harmful genera and enriching beneficial taxa. Direct blood pressure measurements were not performed in this study, and the protective effects were assessed using surrogate markers. Consequently, future studies are required to identify the specific bioactive compounds in the postbiotics through multi-omics analysis, combined with continuous blood pressure monitoring and clinical trials, to validate the antihypertensive potential, efficacy and safety of *L. plantarum* IOB602 and its postbiotics in humans. Taken together, these findings suggest that *L. plantarum* IOB602 and its postbiotic are promising candidates for the development of functional foods or adjunct therapies for hypertension management

## Figures and Tables

**Table 1:** ACE inhibition rate in vitro assay reaction system.

Reagent	Group A/µL	Group B/µL	Group C/µL	Group D/µL
FAPGG working solution	150	150	150	150
37 °C incubation for 5 min				
ACE working solution	10		10	
Sample	10	10		
Sterile nuclease-free water		10		10
Tris-HCL			10	10
37 °C incubation for 5 min				

**Table 2:** Real-time quantitative PCR primer sequence.

Gene	Primer Sequences (5′→3′)
β-actin-F	ATATCGCTGCGCTCGTCGT
β-actin-R	CATACGCACCATCACACCCTGG
PI3K-F	ATACTTGATGTGCCTGACGC
PI3K-	AATCCTCTTCATCGTCTACC
AKT1-F	TCATTGAGCGCACCTTCCAT
AKT1-R	CTCCTGCGGTTTGAGTCCAT
AT1R-F	CACAACCCTCCCAGAAAGTGATC
AT1R-R	GATGATGCTGTAGAGGGTAGGGATC

## Data Availability

The original contributions presented in this study are included in the article. Further inquiries can be directed to the corresponding author.
